# Soft Phase Control of Yield Strength and Impact Toughness in a Q&P-Processed Medium-Carbon Steel

**DOI:** 10.3390/ma19204367

**Published:** 2026-10-17

**Authors:** Roman Mishnev, Yuliya Tkacheva, Kaiming Wu, Rustam Kaibyshev

**Affiliations:** 1 Collaborative Innovation Center for Advanced Steels, Institute of High Technology, Wuhan University of Science and Technology, Wuhan 430081, China; wukaiming@wust.edu.cn; 2 Laboratory for Mechanical Properties of Nanostructured Materials and Superalloys, Belgorod State University, Pobedy 85, Belgorod 308015, Russia; borisova_yu@bsuedu.ru (Y.T.); rustam_kaibyshev@bsuedu.ru (R.K.)

**Keywords:** steels, mechanical properties, product of strength and elongation, impact energy

## Abstract

The yielding behavior and impact toughness of a 0.44 wt.% C steel subjected to quenching and partitioning (Q&P) at 400 °C for 20–1000 s were investigated. The steel has a multiphase microstructure containing blocky retained austenite (RA) and carbide-free bainitic ferrite (BF). Using the continuum composite approach, we show that plastic deformation at the 0.2% offset strain can be described as initiating via dislocation multiplication in RA and BF, while martensite remains elastic. The yield strength (YS) calculated from solid solution and dislocation strengthening agrees with experimental values (1110–1210 MPa) within 3% for all partitioning times. The classical composite model overestimates YS unless only RA and BF are considered as plastically deforming; the combined RA + BF fraction remains nearly constant, explaining the stable YS. The product of strength and elongation reaches 35.3 GPa×% at 300 s. The Charpy impact toughness peaks at 34 J/cm^2^ at 500 s, due to full martensite decomposition, and decreases to ~30 J/cm^2^ at 1000 s, attributed to excessive carbon enrichment of RA (1.63 wt.%), which reduces its plastic accommodation capacity and promotes earlier cleavage initiation.

## 1. Introduction

Advanced high-strength steels (AHSS) are widely used by the automotive industry due to an attractive combination of high ultimate tensile strength (UTS) and ductility, δ, which is characterized by the product of strength and elongation (PSE = UTS × δ) (MPa×%) [1]. To meet the growing demand of the transportation industry, third-generation AHSS with a PSE ranging from 2 × 10^4^ to 4 × 10^4^ MPa×% are under development [1]. A recent comprehensive review confirms that Q&P processing offers considerable potential for automotive applications due to its excellent strength–toughness balance [2]. Important requirements for these steels are a combination of high yield strength (YS) ≥ 1000 MPa with PSE ≥ 3 × 10^4^ MPa×% and sufficient Charpy V-notch (CVN) impact toughness [1]. Strength and ductility are the key properties of advanced high-strength steels (AHSS) for the automotive industry [1]. A superior combination of high yield strength (YS ≥ 1000 MPa) and an excellent product of strength and elongation (PSE ≥ 3 × 10^4^ MPa×%) was achieved in low-alloyed steels with carbon content ≥ 0.4 wt.%C processed by quenching and partitioning (Q&P), placing them among third-generation AHSS [3,4]. These steels typically contain a high-volume fraction of blocky retained austenite (RA), which contributes significantly to their mechanical properties [3,4,5]. It was indicated that the strength of RA could reach ≥1000 MPa due to a combination of a carbon enrichment of ~1.3 wt.% and a high density of lattice dislocations of ~6 × 10^14^ m^−2^ in a 0.44 wt.% C steel [3]. However, the ideal carbon partitioning is often complicated by competing reactions such as bainite formation and carbide precipitation [6]. Detailed microstructural studies confirm that during prolonged partitioning, carbon enrichment of austenite is accompanied by complex reactions that ultimately dictate RA stability [7]. For instance, studies on medium-carbon Q&P steels have shown that adjusting the phase ratio can optimize the balance between strength and ductility by controlling RA morphology and stability [8]. The YS of Q&P steels, characterized by their multiphase microstructure, is often described by the composite model [1,9]:

(1) $$\mathrm{YS}=\sum_{i=1}^{n}\sigma_{i}V_{i}$$

where σ*_i_* is the strength of *i*-constituent and V*_i_* is the volume fraction of *i*-constituent (in %), *n* is the number of structural constituents. Equation (1) is feasible for predicting the theoretical value of YS with high accuracy if the volume fraction of RA is low and its strength is significantly smaller than experimental YS [9]. At the 0.2% offset strain, very high values of the work hardening rate, d*σ*/d*ε* > E/50 (where E is Young’s modulus), were observed in Q&P steels [4,9]. These d*σ*/d*ε* values exceed the limit for the dislocation storage-based mechanism [10]. Therefore, the theoretical calculations of YS by Equation (1), taking into account σ*_i_* values computed by linear addition of different strengthening mechanisms, could be incorrect [10,11]. To address this issue, the continuum composite approach (CCA) has been proposed, which considers sequential yielding of constituents in multiphase microstructures [11,12]. According to CCA, macroscopic yielding occurs when the softest phase undergoes plastic deformation at the 0.2% offset strain, while harder phases remain elastic. The high work hardening rate is then attributed to the gradual elastic-to-plastic transition of these harder phases with increasing strain [11]. The hard phases—primary/tempered martensite (M1) and secondary/fresh martensite (M2)—exhibit significantly higher yield strengths, estimated as ~1700 MPa and ~1500 MPa, respectively, based on quenching and tempering data for similar steel grades [13].

According to established physical yield criteria [14,15], macroscopic yielding begins with the onset of dislocation multiplication via activation of Frank–Read sources. RA is considered a soft phase [9], and the high-volume fraction of blocky-type RA in the 0.44%C Q&P steel [3,4] may provide macroscopic yielding at the 0.2% offset strain by plasticization of this constituent alone. However, low-alloy bainitic steels with a high-volume fraction of carbide-free bainitic ferrite (BF) exhibit YS ranging from 900 to 1200 MPa [1,16,17,18]; therefore, BF should also act as a soft phase contributing to yielding. Systematic studies confirm that processing parameters such as partitioning time critically determine the final phase fractions and RA morphology [19].

Therefore, the present work aims to: (i) apply the CCA to both RA and BF using directly measured microstructural parameters; (ii) calculate the yield strength of each soft phase; and (iii) compare the theoretical YS with experimental values across five partitioning conditions. The novelty is that yielding is controlled jointly by RA and BF, and that the classical composite model overestimates YS unless only these two phases are considered as perfectly plastic at 0.2% offset strain. The Charpy impact toughness is also interpreted within this framework, linking carbon enrichment of RA and BF morphology to cleavage initiation. Mechanical properties and microstructures after partitioning with different durations are considered in a companion article [4].

## 2. Materials and Methods

### 2.1. Material and Q&P Processing

A Fe-0.44C-1.81Si-1.33Mn-0.82Cr-0.28Mo (in wt.%) steel was produced by air induction melting followed by electro-slag remelting. Samples with 3 mm thickness were austenitized at 900 °C for 5 min, followed by quenching at 200 °C for 15 s in a salt bath and then partitioning at temperature 400 °C for *t_p_* = 20, 60, 300, 500, and 1000 s in a salt bath, followed by air cooling to obtain different microstructures. The partitioning durations were selected to capture key stages of microstructure evolution: incomplete carbon partitioning (20 s), optimal RA stabilization (60–300 s), the onset of martensite tempering (500 s), and pronounced carbon enrichment of RA accompanied by bainite refinement (1000 s). A comprehensive description of the phase transformations occurring at each stage is given elsewhere [4]. The critical points of the present steel (M_S_ = 270 °C and M_F_ = 51 °C) are reported in previous work [3] and, therefore, M_S_ < *T_q_* < M_F_, and *T_p_* > M_S_. The quenching temperature was chosen based on Koistinen–Marburger analysis [3,20,21,22,23] to produce ~40% of RA [3].

### 2.2. Mechanical Testing

The flat specimens with a gauge length of 35 mm and a cross-section of 7 × 3 mm^2^ were cut from samples processed by Q&P routes with different partitioning times and then tensioned using the “Instron 5882” testing machine (Illinois ToolWorks Inc., Norwood, MA, USA) at room temperature and an initial loading rate of 1 mm/min in accordance with ASTM E08M-04 standard. Three specimens were tensioned for each partitioning time. A strain gauge extensometer with an accuracy of 0.5% was used to examine the yielding behavior of the specimens. Hardness was measured using a Wolpert Wilson 600 MRD (Illinois ToolWorks Inc., Norwood, MA, USA) tester in accordance with the ASTM E18 standard at room temperature. CVN impact tests were carried out using standard CVN specimens in accordance with the ASTM E-23 standard at room temperature. An Instron SI-1M impact machine with a maximum energy of 450 J (Illinois ToolWorks Inc., Norwood, MA, USA), followed by the Instron Dynatup Impulse data acquisition system, was used. Two CVN specimens were tested for each partitioning time.

### 2.3. Microstructural Characterization

The scanning electron microscope (SEM) observations and electron backscatter diffraction (EBSD) analysis, providing reconstruction of misorientation maps with 100 × 100 µm^2^ in area and a scan step of 0.15 µm, were carried out using a Quanta 600 FEG SEM (FEI Corporation, Hillsboro, OR, USA) operated at 20 kV and equipped with an EDAX Velocity™ camera (EDAX Inc., Mahwah, NJ, USA). These maps were used to calculate the dimensions of RA blocks. Dislocation density in RA, BF, and martensite was calculated using Kernel average misorientation (KAM), θ_KAM_. A JEOL JEM-2100 transmission electron microscope (TEM) (JEOL Ltd., Tokyo, Japan) was used to evaluate dimensions of film-like RA and thickness of laths delimited by LABs with misorientation < 2°.

The volume fraction of RA was determined by means of magnetic saturation measurements using a Fischer Feritscope FMP30 (Helmut Fischer Gmbh, Sindelfingen, Germany) and X-ray analysis using a Rigaku Ultima IV diffractometer (Rigaku Co., Tokyo, Japan) with a step size of 0.02 deg, 40 kV and 40 mA, and a 2*θ* Bragg angle range from 30 to 135°. X’pert High Score Plus software (2.1) was used to calculate the volume fraction of RA according to the peak intensities for the fcc lattice [24,25].

The uncertainties of the microstructural parameters are estimated as follows: phase fractions ± 1–2 vol.%, carbon concentration in RA ± 0.05 wt.%, dislocation density ± 0.5 × 10^14^ m^−2^, and microstructural dimensions ± 0.02–0.05 μm. Other details of the material, Q&P processing, and the mechanical characterization and structural characterization techniques were reported previously [3,4,26].

## 3. Results

### 3.1. Microstructure

**Figure 1:**
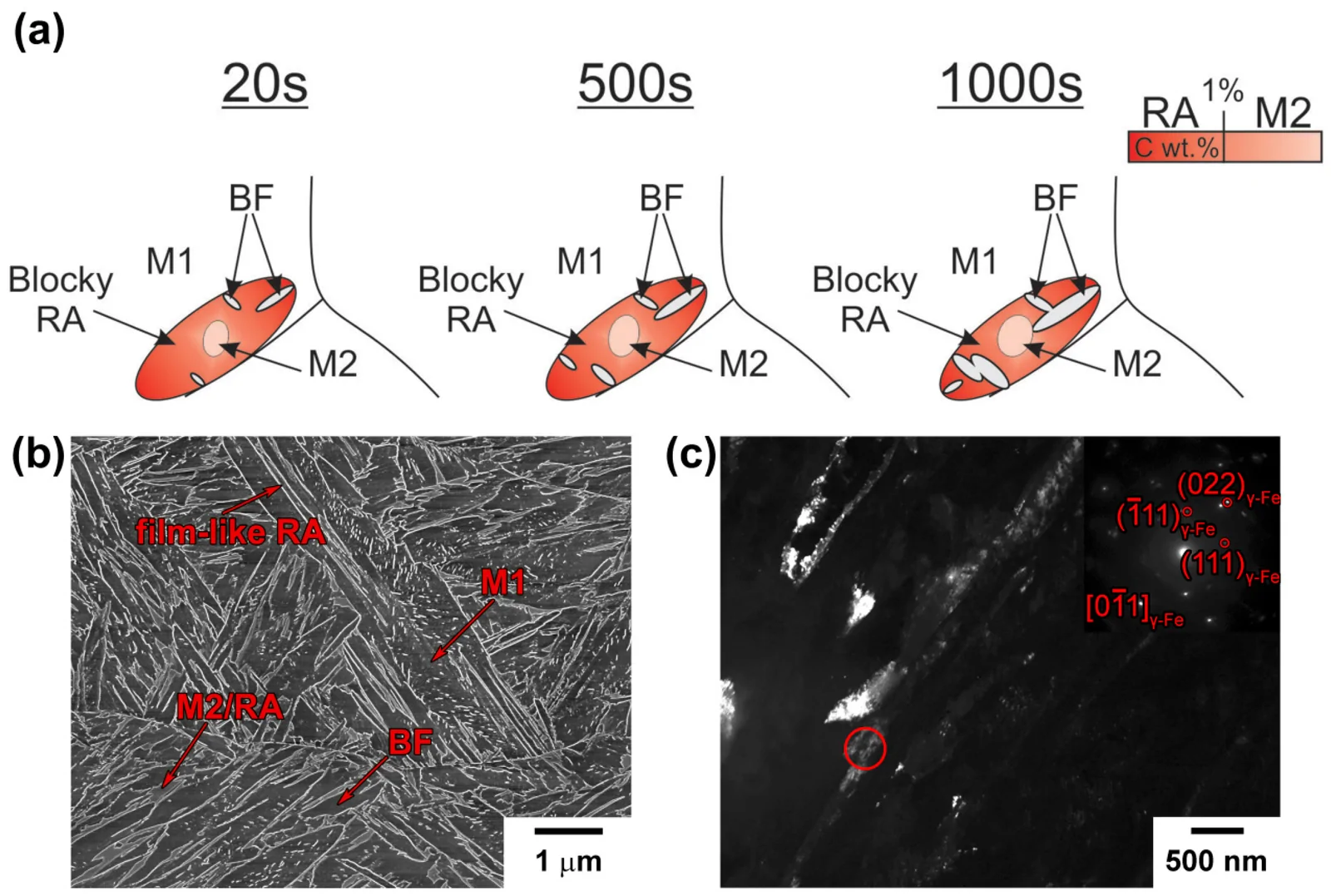
Schematic illustrations of the effect of partitioning time on the microstructure of the steel after Q&P processing [12,26] (**a**), SEM micrograph after partitioning time 300 s (**b**), dark-field TEM image showing RA lamellas after partitioning for 60 s (**c**).

The microstructure of the Fe-0.44C steel comprises primary/tempered martensite, secondary/fresh martensite, blocky RA, film-like RA, carbide-free bainitic ferrite (BF), and carbide particles located within the primary martensite [3,4]. Table 1 summarizes the phase fractions as a function of partitioning time. Although the volume fraction of retained austenite varies with partitioning time, the combined fraction of RA and BF remains almost unchanged (Table 1). It is worth noting that blocks of RA consist of RA lamellas subdivided by sheaves of BF (Figure 1) [4]. Additionally, secondary martensite evolved in core areas of the RA upon final cooling [4]. Film-like RA with a thickness of 160 nm is located between laths and blocks of primary martensite [4]. Figure 1c presents a TEM image showing a typical film-like RA grain with a thickness of approximately 160 nm, located between two laths of primary martensite [26]. This morphology was consistently observed across all partitioning conditions. The volume fraction of film-like RA was estimated to be about 4%, which is remarkably lower than that of blocky RA. It should be noted that the V_RA_ values reported in Table 1 represent the total RA fraction, including both blocky and film-like morphologies.

### 3.2. Mechanical Properties

**Figure 2:**
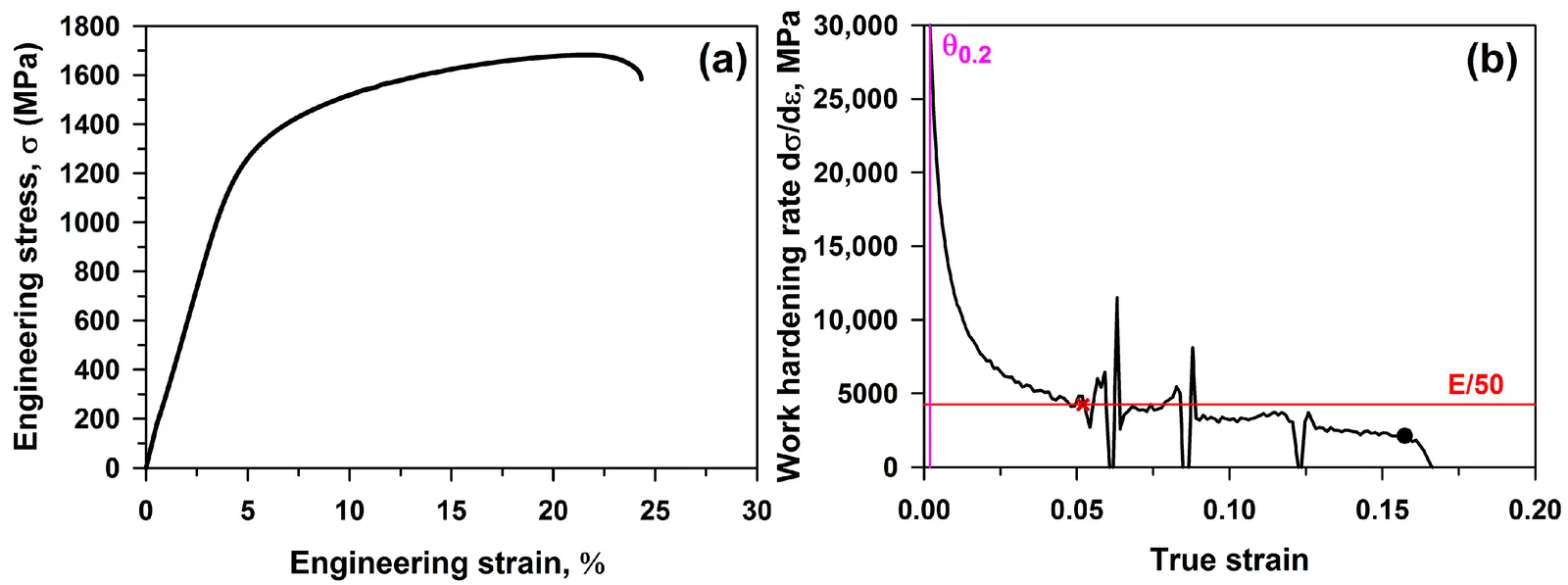
The stress–strain curve (**a**) and the work hardening rate vs. strain curve (**b**) after *t*_p_ = 60 s [4].

Typical engineering stress–strain curve and the work hardening rate vs. strain curve for the sample subjected to partitioning for 60 s are shown in Figure 2. The effect of *t*_p_ on YS and the work hardening rate at the 0.2% offset strain is detailed in Table 2 and Table 3. The parameters in Table 2 represent the average of tests, with the standard deviation provided. Parameters in Table 3 were derived from the individual stress–strain curves. All σ-ε curves exhibit continuous yielding followed by an extended work hardening stage with the d*σ*/d*ε* > E/50 (Figure 2, Table 3). Therefore, the 0.2% offset strain is used here as the macroscopic definition of the elastic-plastic transition [11,14,15]. The high work hardening rates suggest that the CCA is applicable for describing the macroscopic yielding behavior of this steel [26].

**Figure 3:**
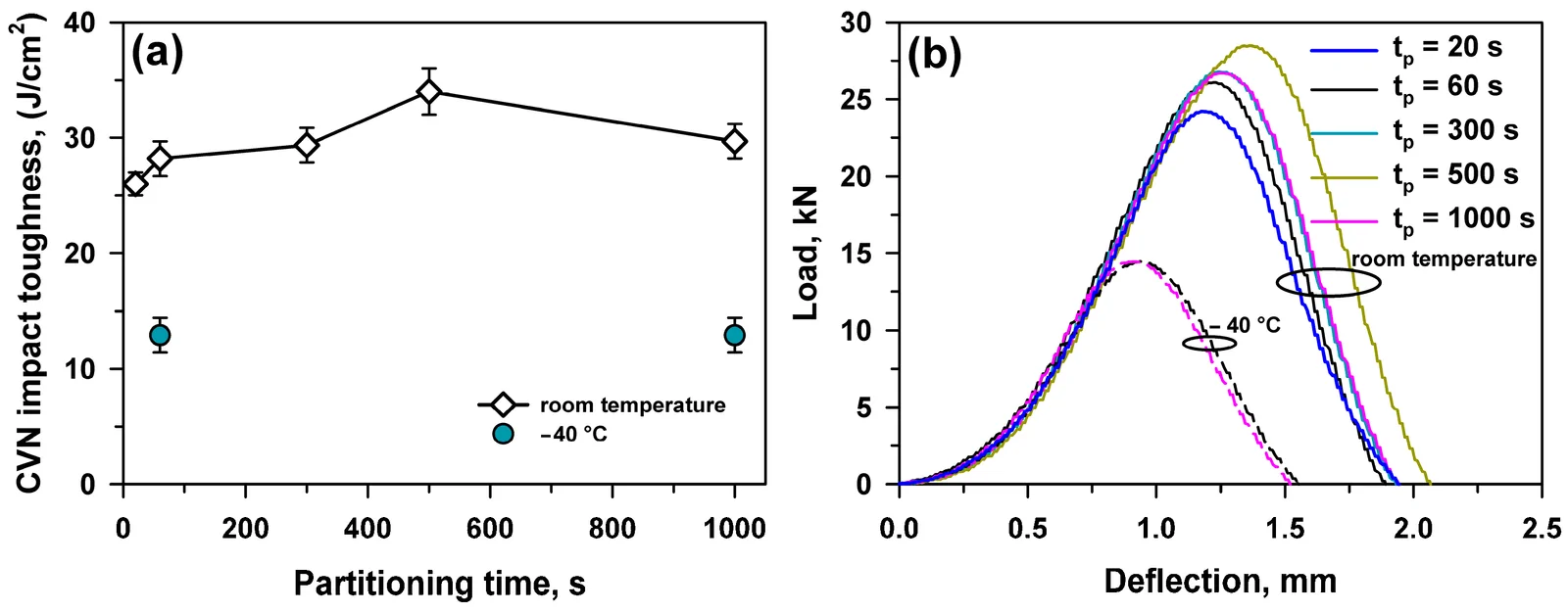
Effect of partitioning time on impact toughness (**a**) and load-deflection curves (**b**) at room temperature and −40 °C.

CVN impact toughness at room temperature increases with increasing partitioning time up to 500 s (Table 2). Further partitioning to 1000 s decreases impact toughness (Table 2). Therefore, no relation between the volume fraction of RA and impact toughness takes place. Impact toughness tends to increase with increasing volume fraction of transition carbides and, therefore, depletion of carbon from martensite is a key parameter for CVN impact toughness. A single type of load-deflection curve was observed [13,21,22,23,27], where only the maximum load, P_M_, is clearly defined. The increase in impact toughness is attributed to the rise in P_M_ value (Figure 3, Table 2). No points associated with general yielding, transition to unstable crack propagation, and crack arrest were revealed. This impact behavior is typical for Q&P steels and is characterized by relatively high energies dissipated for the initiation of a crack with critical dimension, E_i_, and at the stage of crack propagation, E_p_ [13,21,22,23,27]. The values of E_i_ and E_p_ were nearly identical. The E_p_ energy ranging from 13 to 17 J is relatively high, and no well-defined unstable crack propagation stage is distinguished, as in other steels with Si ≥ 1.5 wt.% [13,21,22,23,27]. At −40 °C, the steel after Q&P processing exhibits significantly smaller CVN impact toughness than at ambient temperature.

### 3.3. Fractography

**Figure 4:**
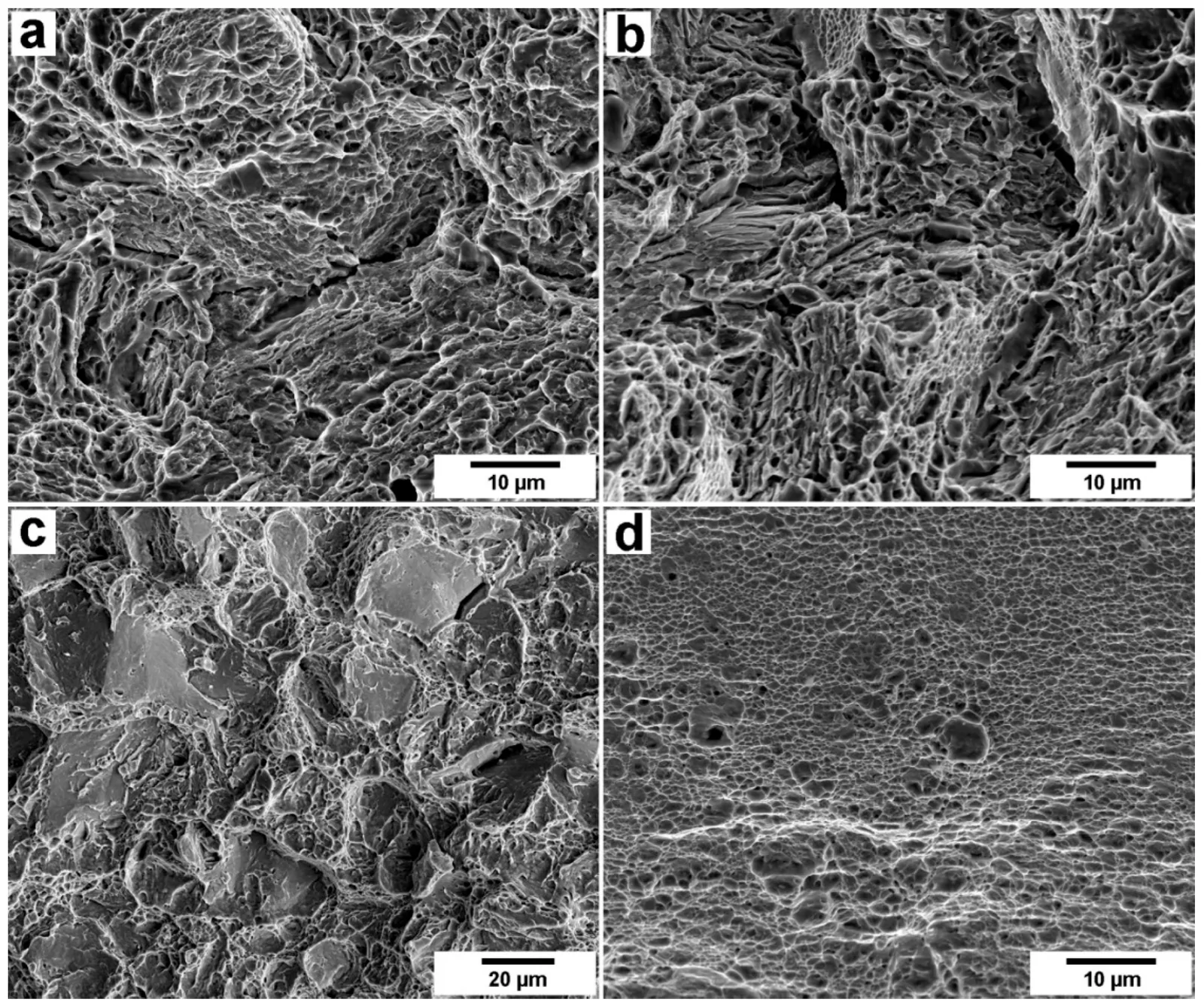
Fracture surfaces of crack initiation areas in specimens tensioned up to failure after 60 (**a**), 300 s (**b**) and 1000 s (**c**) partitioning and crack propagation area after 300 s partitioning (**d**).

Figure 4 shows that crack initiation occurs by quasi-cleavage fracture during tension. Partitioning strongly affects the mechanism of crack nucleation. After partitioning, with times ranging from 20 to 300 s, the crack initiation process occurs by mixed fracture (Figure 4a). Cleavage stopping at block boundaries [13,21] leads to crack nucleation, but dimple transgranular fracture also gives a significant contribution to microcrack growth (Figure 4a). The role of intragranular fracture in the growth of microcracks is minor (Figure 4a). After 300 s of partitioning, intragranular fracture is suppressed, and cleavage propagates coherently across entire blocks within the martensitic constituents (Figure 4b). With partitioning times ≥ 500 s, the cleavage propagates through whole PAGs (Figure 4c), which greatly decreases fracture stress and leads to premature fracture (See Section 3.2) [13,21]. The portion of ductile fracture decreases in comparison with partitioning with shorter times, but remains significant (Figure 4c). Crack propagation near sample edges occurs in a ductile manner at all partitioning conditions (Figure 4d).

**Figure 5:**
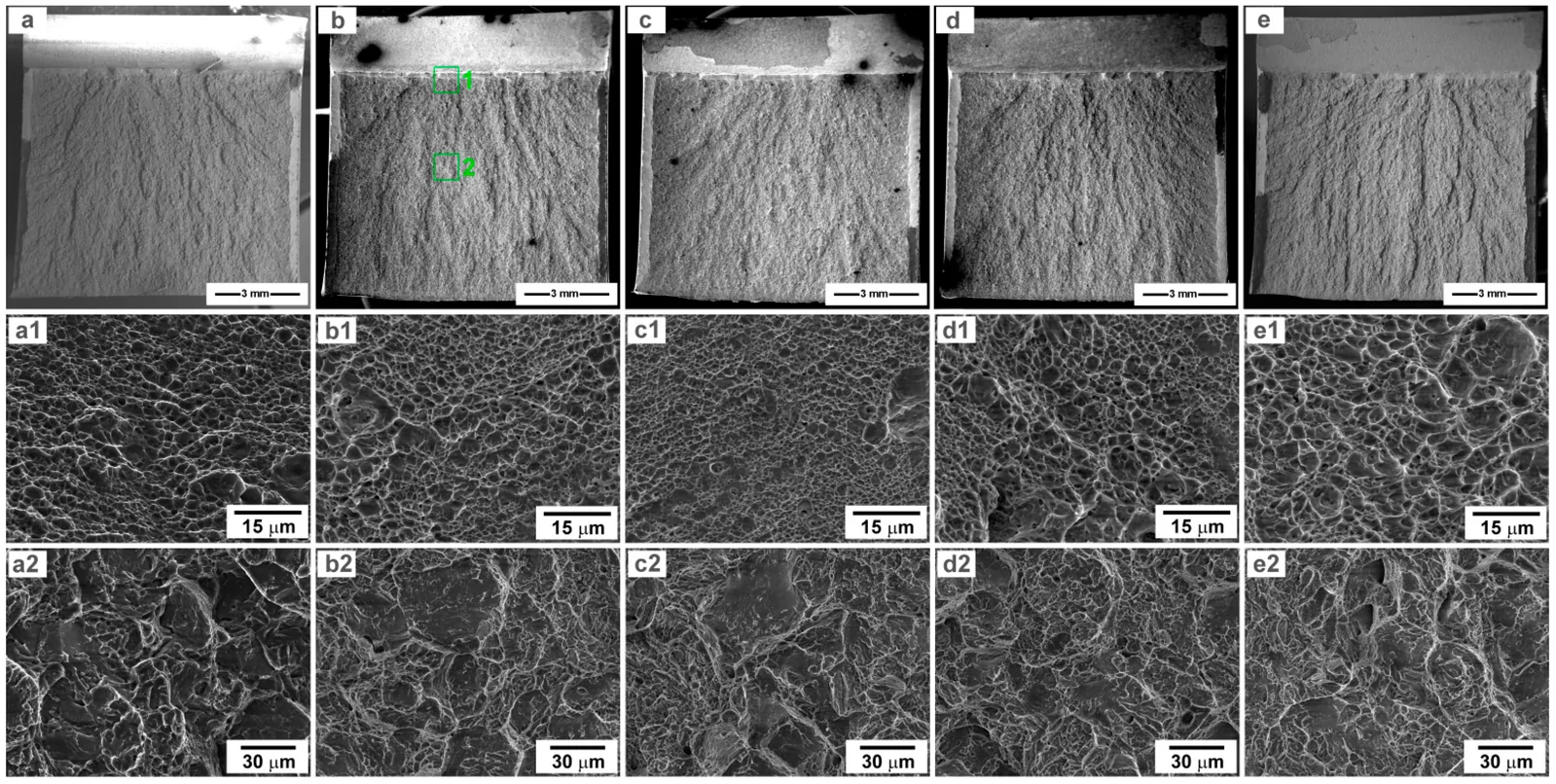
Low-magnification general view (**a**–**e**) and high-magnification fractographs of IZ (**a1**–**e1**), FCPZ (**a2**–**e2**); t_p_ = 20 s (**a**–**a2**), t_p_ = 60 s (**b**–**b2**), t_p_ = 300 s (**c**–**c2**), t_p_ = 500 s (**d**–**d2**), t_p_ = 1000 s (**e**–**e2**) of the Fe-0.44C steel.

Figure 5 shows fracture surfaces of CVN specimens tested at room temperature. The fracture surfaces consist of an initiation zone (IZ), fast crack propagation/fibrous zone (FCPZ), and shear-lip zone (SLZ) (Figure 5a–d) [13,21,22,28,29,30]. IZ and FCPZ are distinctly distinguished fracture mechanisms and are presented in Figure 5a1–e1 and Figure 5a2–e2, respectively. SLZ is located at the lateral edges, and no remarkable difference in fractography between IZ and SLZ was found in previous work [21] and the present study. A fully dimpled fracture was observed in the SLZ zone [21], as in the present study. F. Zia-Ebrahimi et al. [28] interpreted crack propagation in this zone in terms of the arrest of unstable crack propagation. However, it was recently assumed [21] that the concurrent formation of FCPZ and SLZ takes place, taking into account the rectangular lateral surfaces, and the aforementioned shape of the load displacement curves with one P_M_ well-defined point and relatively high E_p_ values. Therefore, fast crack propagation partially occurs in a fully ductile manner.

Coarse and deep dimples alternate with shallow and fine dimples are observed in the IZ (Figure 5a1–e1), and increasing partitioning time from 20 to 500 s expands this zone (Table 2). After partitioning for 1000 s, the dimension of the IZ decreases (Table 2). Mixed fracture takes place in the FCPZ. Quasi-cleavage fracture is the main mechanism in this zone. After 20 s partitioning, the PAGs play a role in the effective grain size for brittle fracture (Figure 5a2) [13,31,32]. Boundaries of packets and blocks play the role of obstacles for cleavage propagation (Figure 5a2) [13,31,32], occasionally. Packets become predominated as the effective grain size for brittle fracture [13,31,32] after 300 s partitioning (Figure 5b2,c2). After 500 s partitioning, the effective grain size for brittle fracture [13,31,32] could not be distinguished due to a high proportion of coarse dimples at the fracture surface in the FCPZ (Figure 5d2,e2). Therefore, refinement of the effective grain size for brittle fracture takes place with partitioning. In addition, the portion of dimple fracture increases with increasing partitioning time. Areas of coarse shallow dimples alternate with areas of quasi-cleavage fracture. Fine dimples are observed at tear ridges located at the boundaries of PAGs. The fracture becomes more pronounced with partitioning.

## 4. Discussion

### 4.1. Effect of Partitioning on YS

The effect of *t*_p_ on the work hardening rate at the 0.2% offset strain is detailed in Table 3. All σ-ε curves exhibit continuous yielding followed by an extended work hardening stage with the $\frac{d\sigma }{d\varepsilon }>\frac{E}{50}$ (Figure 2, Table 3). Therefore, the 0.2% offset strain clearly defines the elastic–plastic transition [11,12,14,15]. The high work hardening rates (Table 3) suggest that the CCA is applicable for describing the macroscopic yielding behavior of this steel.

The strength of RA can be described by a linear superposition of strengthening mechanisms expressed as [3,9,33,34]:

*σ*_RA_ = *σ*_0_ + *σ*_SS_ + *σ*_HP_ + *σ*_disl_ (2)

where *σ*_0_ = 64 MPa is the lattice friction stress in γ-iron [35], *σ*_SS_ is the solid solution strengthening, *σ*_disl_ is the dislocation strengthening, and *σ*_HP_ is the grain boundary strengthening. Here, RA includes both blocky and film-like RA, and Equation (2) is applied to this total RA fraction, since any austenite surviving to room temperature is carbon-enriched and heavily dislocated, giving both morphologies similar strength. The solid solution strengthening component follows the relationship [35,36]:

*σ*_SS_ = 356.5 × (wt.%C) + 20.1 × (wt.%Si) + 3.7 × (wt.%Cr) + 14.6 × (wt.%Mo) (3)

The manganese was excluded from the calculations due to its negligible weakening effect on the *σ*_SS_. The contribution of manganese to solid solution strengthening was estimated using the coefficient from [35] (≈4 MPa per wt.%). With a Mn content of 1.33 wt.%, the contribution is approximately 5.3 MPa—less than 0.5% of the experimental YS. The average concentrations of substitutional solutes were taken from the bulk steel composition, as Q&P processing does not induce significant partitioning of these elements [1,9]. As shown in Table 3, the solid solution strengthening provides the dominant contribution to RA strength, with its magnitude increasing proportionally with carbon concentration in RA. It is important to note that this increase has a good correlation with an increase in YS, regardless of the volume fraction of RA (Table 1 and Table 3).

The *σ*_disl_ contribution can be described by a Taylor-type relationship [34]:

*σ*_disl_ = *αMGbρ*^0.5^ (4)

where *α* is the dislocation strengthening, *M* = 3.06 is the Taylor factor taken from EBSD data presented in companion work [4], *G* = 75 GPa is the shear modulus [36], *b* = 0.25 nm is the magnitude of the Burgers vector [3,9,36], and *ρ* is the lattice dislocation density (Table 1). The *α* = 0.3 was adopted for the calculation of the *σ*_disl_ value, taking into account the nearly uniform distribution of forest dislocations in blocky RA [4,37]. The high strength of the blocky RA is attributed to the superposition of carbon enrichment and high dislocation density (Table 3). The calculated strength of retained austenite (RA) excluding grain boundary strengthening, $\sigma_{\mathrm{RA}}^{\mathrm{nHP}}$, matches well the experimental yield strength (YS) values (Table 3).

Contribution of the grain boundary strengthening to overall YS was evaluated by the Hall–Petch-type relationship [34]:

*σ*_HP_ = K_HP_*D*^−0.5^ (5)

where K_HP_ = 160 MPa × μm^0.5^ is the Hall–Petch constant taken from recent work [13,36]; and *D* is the effective grain size. Following the approach of Zhang et al. [38] for lamellar microstructures, the effective grain size D is taken as twice the thickness of RA lamellas (D = 2d_RA_). This convention accounts for dislocation pile-ups across two adjacent lamellae, which represent the fundamental unit for Hall–Petch strengthening in lamellar structures. In the present steel, the subdivision of blocky RA into lamellae by bainitic ferrite sheaves (Figure 1) justifies this lamellar approximation. The *σ*_HP_ values and theoretical strength of RA, $\sigma_{\mathrm{RA}}^{\mathrm{HP}}$, calculated under the presumption that the passage of dislocation multiplication occurs from one lamella to the other, are presented in Table 3. It is seen (Table 3) that consideration of the grain boundary strengthening leads to overestimation of experimental YS.

Another soft phase is BF, which exhibits similar behavior and shows good agreement in calculations without the Hall–Petch contribution. As shown previously, the blocky RA is subdivided into alternating lamellae of RA and sheaves of carbide-free bainitic ferrite (BF) during partitioning. BF is also considered a “soft” phase (σ ≈ 900–1200 MPa [1,3,17,18]) and may contribute to macroscopic yielding together with RA. To verify this, we calculate the theoretical strength of BF using an analogous approach to Equation (2), but accounting for the bcc lattice and its specific strengthening mechanisms.

Solid solution strengthening in BF is calculated using Pickering’s empirical equation [39]:

*σ*_SS_ = 32Mn + 83Si + 11Mo − 31Cr + 5544C_ss_ (6)

where Mn, Si, Mo, and Cr are the bulk compositions (in wt.%), and C_ss_ = 0.1 wt.% is the equilibrium carbon content in bainitic ferrite at 400 °C, confirmed for Si-rich steels [40,41]. Substituting the steel composition (Mn = 1.33, Si = 1.81, Mo = 0.28, Cr = 0.82) gives *σ*_ss_ = 724 MPa.

The dislocation strengthening in BF, calculated using Equation (4) with *G* = 81.6 GPa, was *σ*_disl_ = 348 MPa [12,42,43]. For the bcc lattice, we use dislocation strengthening factor α = 0.24 (typical for low-alloy multiphase steels [38,44,45,46]), dislocation density in BF ρ_BF_ = 5.4 × 10^14^ m^−2^ (measured by KAM, consistent with [3,4]), and Taylor factor M = 3.06 (from EBSD). Lattice friction for α-iron is *σ*_0_ = 45 MPa [35,36,43].

Thus, the calculated strength of BF without grain boundary contribution (i.e., assuming dislocations do not cross BF/BF or BF/RA boundaries at yielding) is = 1118 MPa. This value (1118 MPa) is very close to the experimental YS (1110–1210 MPa, Table 2) and to the calculated RA strength (1073–1183 MPa, Table 3). It confirms that plastic deformation at the 0.2% offset strain is initiated concurrently in RA lamellae and BF sheaves, while the hard martensitic phases (M1, M2) remain elastic.

For completeness, the grain boundary contribution in BF was evaluated using the Hall–Petch relation (Equation (5)) with K_HP_ = 310 MPa × μm^0.5^ [18,47] and D = 2d_BF_ (Table 1). Including $\sigma_{\mathrm{BF}}^{\mathrm{HP}}$ overestimates the YS by 15–25% (Table 3), indicating that dislocation transmission across boundaries does not occur at the yield point. Therefore, the combined deformation of RA and BF using only solid solution and dislocation strengthening (without Hall–Petch) fully describes the experimental yield strength of the steel.

The composite rule (Equation (1)) can predict YS (Table 3) if only RA and BF are treated as plastically deforming at 0.2% offset strain, without dislocation transfer across their boundaries. Their volume fractions satisfy V_RA_ + V_BF_ = 1. The theoretical YS $\sigma_{0.2}^{\mathrm{theor}}$ was calculated by normalizing the RA and BF fractions to their combined fraction:

(7) $$\sigma_{0.2}^{\mathrm{theor}}=\sigma_{\mathrm{RA}}^{\mathrm{nHP}}\cdot \frac{V_{\mathrm{RA}}}{V_{\mathrm{RA}}+V_{\mathrm{BF}}}+\sigma_{\mathrm{BF}}^{\mathrm{nHP}}\cdot \frac{V_{\mathrm{BF}}}{V_{\mathrm{RA}}+V_{\mathrm{BF}}}$$

where $\sigma_{\mathrm{RA}}^{\mathrm{nHP}}$ and $\sigma_{\mathrm{BF}}^{\mathrm{nHP}}$ are the sums of solid solution and dislocation strengthening for RA and BF, respectively. Martensite (M1, M2) remains elastic at yielding. The resulting theoretical YS, $\sigma_{0.2}^{\mathrm{theor}}$, matches the experimental values (Table 3). Thus, Equation (1) works when only the perfectly plastic phases are summed. It should be noted that in the present CCA analysis, RA and BF are treated as perfectly plastic at the 0.2% offset strain. This assumption implies that once these soft phases reach their yield point, they deform plastically without significant additional strain hardening within the narrow strain interval used to define the offset. This simplification is justified for the following reasons. First, the CCA is applied here to describe the onset of macroscopic yielding, not the subsequent work hardening stage. At the 0.2% offset strain, plastic deformation in the soft phases has just begun, and their intrinsic work hardening is small compared to the elastic constraint imposed by the surrounding hard martensitic constituents. Second, the initial dislocation density in RA and BF is already high (≈7–8 × 10^14^ m^−2^ for RA and 5.4 × 10^14^ m^−2^ for BF), and the corresponding Taylor-type strengthening contribution is explicitly included in the calculated yield strength. As a result, these phases are already in a strain-hardened state, and additional hardening over the small plastic strain up to the 0.2% offset is negligible. Third, the good agreement between the calculated yield strength using constant σ_RA_ and σ_BF_ values and the experimental YS for all partitioning conditions supports the validity of this assumption.

This agreement is consistent with the interpretation that yielding starts by plastic flow in soft RA and BF, while hard martensite stays elastic. By contrast, σ_c_—obtained by applying Equation (1) to all phases (including M1, M2) as if they were plastic from the start—gives an upper-bound estimate that greatly exceeds the actual YS. This overestimation occurs because the hard phases do not yield at the same time as the soft ones; the true behavior involves a gradual elastic–plastic transition.

Although the volume fraction of retained austenite varies with partitioning time, the combined fraction of RA and BF remains almost unchanged. This indicates that the total amount of soft phases (RA + BF) is relatively stable (Table 1), while the ratio between them changes due to bainitic transformation and subsequent austenite growth. Thus, even though the retained austenite fraction is low at 500 and 1000 s, the combined soft phase fraction (RA + BF) remains high (Table 1), which yields consistent agreement for all partitioning conditions and supports the conclusion that macroscopic yielding is provided solely by dislocation multiplication in RA and BF, in accordance with the physical yield criterion [14,15].

A sensitivity analysis of the calculated yield strength with respect to the uncertainties in the microstructural parameters was performed. Varying the dislocation density by ±10% changes the calculated RA strength by ~3–5%, which is within the experimental scatter of YS. Varying the carbon content in RA by ±0.05 wt.% changes *σ*_SS(RA)_ ~ 18 MPa, which is also within the scatter. The combined effect of these uncertainties on the calculated YS is less than 4%, consistent with the agreement between theory and experiment reported in this work.

### 4.2. Relation Between PSE and the CVN Impact Toughness

Inspection of the experimental data shows that a correlation between the PSE value and impact toughness takes place with increasing partitioning time from 20 to 300 s (Table 2). The volume fraction of blocky RA, dimensions of RA blocks, and carbon enrichment affect ductility and the PSE value, as evidenced by the data in Table 1, Table 2 and Table 3. For instance, the highest PSE of 35 310 MPa×% at 300 s coincides with an RA fraction of 16%, a maximum RA block size D_RA_ = 3.2 μm, and moderate carbon enrichment (*σ*_SS(RA)_ ≈ 599 MPa). If grains of blocky RA are coarse and a high-volume fraction of RA can transform to strain-induced martensite during tension, the PSE is high. A decrease in the volume fraction of RA with increasing partitioning time from 60 to 300 s correlates with increasing ductility and the PSE. Subdivision of RA blocks by thin bainitic sheaves and enrichment of carbon in all RA structural elements promote ductility and provide the highest PSE. Further refinement of RA blocks (D_RA_ = 1.0 μm at 500 s) and increasing carbon enrichment hinder its transformation to strain-induced martensite [48], which decreases the PSE. It is worth noting that ductility and the PSE remain high after 500 and 1000 s partitioning, with PSE > 25,000 MPa×%. The decrease in the PSE from 35,310 MPa×% at 300 s to 25,740 MPa×% at 1000 s is attributed to premature fracture due to decreased fracture stress. Therefore, the role of RA consists in preventing premature fracture due to microcrack nucleation. In general, the CVN impact toughness is controlled by the martensitic matrix, since an insignificant effect of RA characteristics on ductility was found.

High fracture toughness is attributed to the large stress for brittle fracture, which can be expressed by the following relationship [13,21,27,49,50]:

(8) $$\sigma_{F}=1.41\sqrt{\frac{{2\mathrm{E\gamma}}_{s}}{\pi \left(1-\nu^{2}\right)}}d_{\mathrm{eff}}^{-1/2}$$

where *E* = 212 GPa is Young’s modulus, *γ_s_* is the surface energy of the cleavage plane for the martensite structure, *υ* = 0.293 is Poisson’s ratio, and *d_eff_* is the effective grain size for the fracture. Depletion of carbon from martensite highly increases the *γ_s_* value. Increasing the volume fraction of transition carbides indicates a deep carbon depletion of martensite. Highest CVN impact toughness is attained after 500 s partitioning after full precipitation of transition carbides; this provides the highest *σ_F_* that requires the highest dimension of critical crack susceptible to fast propagation. Consequently, the proportion of ductile fracture is maximized, resulting in good fracture toughness.

The decrease in CVN impact toughness observed between 500 and 1000 s of partitioning can be rationalized within the framework of the CCA. At 1000 s, RA becomes substantially enriched in carbon (1.63 wt.%, Table 1), which raises its solid solution strengthening contribution (*σ*_SS(RA)_ = 688 MPa, compared to ~600 MPa at 20–300 s) and, consequently, its yield strength. The BF sheaves also become thinner (d_BF_ decreases from 0.6 μm at 500 s to 0.3 μm at 1000 s, Table 1). As a result, the soft phases lose some of their capacity to accommodate local stress concentrations ahead of a propagating cleavage crack. This is consistent with the observed reduction in the initiation zone size (from 0.23 to 0.18 mm, Table 2) and the increase in quasi-cleavage area in the FCPZ (Figure 5e2). Thus, while full carbon depletion of martensite (after 500 s) maximizes the cleavage surface energy *γ_s_* and provides the highest CVN impact toughness, further carbon enrichment of RA and refinement of BF at 1000 s shift the deformation behavior towards earlier local fracture initiation, reducing the overall toughness.

## 5. Conclusions

The yielding behavior and impact toughness of a Fe-0.44C-1.81Si-1.33Mn-0.82Cr-0.28Mo steel processed by quenching and partitioning (Q&P) at 400 °C for 20–1000 s were investigated. The following conclusions can be drawn:

1. The yield strength of the Fe-0.44C Q&P steel is accurately described by the strength of the soft phases—retained austenite (RA) and carbide-free bainitic ferrite (BF)—calculated as *σ* = *σ*_0_ + *σ*_SS_ + *σ*_disl_. The calculated values agree with experimental YS within 3% for all partitioning times (20–1000 s).
2. The classical composite model (Equation (1)) strongly overestimates YS when all phases are assumed plastic, but correctly predicts YS when only RA and BF are treated as plastically deforming at 0.2% offset strain. Although the RA fraction varies, the combined RA + BF fraction remains nearly constant, explaining the consistent YS across conditions. Including Hall–Petch strengthening overestimates YS and is therefore excluded. These results suggest that macroscopic yielding is governed primarily by dislocation multiplication in RA and BF, in accordance with the continuum composite approach and physical yield criteria.
3. The Charpy V-notch impact toughness reaches a maximum of ~34 J/cm^2^ at 500 s, due to full martensite decomposition and precipitation of transition carbides that increase the cleavage surface energy; the subsequent decrease to ~30 J/cm^2^ at 1000 s is attributed to excessive carbon enrichment of RA (1.63 wt.%) and changes in BF morphology, which reduce the plastic accommodation capacity of the soft phases and promote earlier cleavage initiation.

## Figures and Tables

**Table 1:** Effect of partitioning time on the phase volume fractions (RA, M1, M2, BF), dimensions of RA blocks and lamellas, thickness of BF sheaves, and dislocation density in RA [4].

Partitioning Time, s	20	60	300	500	1000
V_RA_, %	12	18	16	8	10
V_BF_, %	6	7	9	15	13
V_M1_, %	70	70	70	72	70
V_M2_, %	12	5	5	5	7
D_RA_, μm	1.9	2.6	3.2	1	1.6
d_RA_, μm	0.2	0.2	0.24	0.12	0.24
d_BF_, μm	0.4	0.45	0.35	0.6	0.3
*ρ*_RA,_ × 10^14^ m^−2^	7.7	8.2	8.4	7.0	8.3

**Table 2:** Mechanical properties of the Fe-0.44%C steel after partitioning at different times.

Partitioning Time, s	20	60	300	500	1000
Hardness, HRC	49 ± 2	48 ± 2	48 ± 2	50 ± 2	49 ± 2
YS, MPa	1110 ± 10	1120 ± 10	1150 ± 10	1170 ± 10	1210 ± 15
UTS, MPa	1790 ± 15	1680 ± 10	1650 ± 10	1660 ± 10	1650 ± 10
Elongation-to- failure, %	13.7 ± 0.5	18.9 ± 0.8	21.4 ± 1.0	19.1 ± 0.8	15.6 ± 0.5
Uniform elongation, %	10.2 ± 0.3	17.3 ± 0.5	17.5 ± 0.6	17.1 ± 0.5	14.7 ± 0.3
PSE, MPa×%	24,523	31,752	35,310	31,706	25,740
CVN at 20 °C, J/cm^2^	26.0 ± 1	28.2 ± 1.5	29.4 ± 1.5	34.0 ± 2	29.7 ± 1.5
CVN at −40 °C, J/cm^2^	–	12.9 ± 1	–	–	12.9 ± 1
Initiation zone (20 °C), mm	0.12	0.14	0.18	0.23	0.18

**Table 3:** The effect of partitioning time on YS [4], work hardening rate at the 0.2% offset strain, *θ*_0.2_ = d*σ*/d*ε* and carbon concentration in this phase [26], *C*_RA_, showed along with the impact of *t_p_* on the contributions of various strengthening mechanisms to the strength of RA and BF. The values were calculated from individual stress–strain curves.

Partitioning Time, s		20	60	300	500	1000
Experimental data	*θ*_0.2_ = d*σ*/d*ε*	24,047	30,190	28,500	15,622	13,192
	*θ*_0.2_ as a portion of Young’s modulus	E/8.5	E/6.8	E/7.2	E/13.1	E/15.5
	*C*_RA_, wt.%	1.37	1.38	1.38	1.43	1.63
Calculated strengthening contributions	*σ*_SS(RA)_ *+ σ*_0_, MPa	595	599	599	617	688
	*σ*_disl(RA)_*,* MPa	478	493	499	455	495
	*σ*_HP(RA)_, MPa	252	252	231	326	231
	*σ*_SS(BF) )_ *+ σ*_0_, MPa	770	770	770	770	770
	*σ*_disl(BF)_*,* MPa	348	348	348	348	348
	*σ*_HP(BF)_, MPa	346	326	370	282	400
	$\sigma_{\mathrm{RA}}^{\mathrm{nHP}}$, MPa	1073	1092	1098	1072	1183
	$\sigma_{\mathrm{RA}}^{\mathrm{HP}}$, MPa	1325	1344	1329	1398	1414
	$\sigma_{\mathrm{BF}}^{\mathrm{nHP}}$, MPa	1118	1118	1118	1118	1118
	$\sigma_{\mathrm{BF}}^{\mathrm{HP}}$, MPa	1464	1444	1488	1400	1518
	$\sigma_{0.2}^{\mathrm{theor}}(\mathrm{RA}+\mathrm{BF})$, MPa	1103	1111	1111	1088	1155
	*σ_c_*(*M*1 + *M*2 + *RA* + *BF*), MPa	1617	1608	1611	1621	1634

## Data Availability

The original contributions presented in this study are included in the article. Further inquiries can be directed to the corresponding author.
